# Books and Authors

**Published:** 1911-02

**Authors:** 


### BOOKS AND AUTHORS

**Transactions of the American Association of Genitourinary Surgeons.** Vols. 3 and 4. Twenty-second and twenty-third annual meetings held at Hot Springs, Va., May 1 and 2, 1908, and at Mount Pocono, Pa., May 31, and June 1, 1909. Edward L. Keyes, Jr., M.D., Secretary.

I. The third volume of these transactions contains the proceedings of the twenty-second annual meeting of the association held at Hot Springs, Va., May 1 and 2, 1908, which was presided over by Dr. John Vander Poel, of New York. The book, bound in paper, is filled with interesting material consisting of papers and discussions thereon. We may mention two in particular without prejudice to the others. One by Bransford Lewis on "individuality in the development of the modern microscope;" and one by Hugh H. Young entitled "Remarks on a fatal case after one hundred and twenty-eight consecutive successful cases of perineal prostatectomy."

II. The fourth volume records the proceedings of the twenty-third annual meeting, which was held at Mount Pocono, Pa., May 31, and June 1, 1909, under the presidency of Dr. Hugh H. Young of Baltimore. This volume, like the preceding one, teems with interesting papers and discussions, though it is somewhat larger, being increased in size by 148 pages. Many of the illustrations are graphic, elaborating cases or instruments in an excellent manner. The paper by Dr. E. Wood Ruggles, of Rochester, entitled "A new method of treatment of phagedenic chancroid and chancre by means of hot air," deserves special mention. The paper of Dr. Hugh Cabot, of Boston, on the "Treatment of stricture of the bulbar portion of the urethra by partial or complete resection" is one of importance and the subject is well presented.

---

**The Practice of Medicine.** A guide to the nature, discrimination and management of disease. By **A. O. J. Kelly,** M.D., Assistant Professor of Medicine, University of Pennsylvania. Octavo, 949 pages, illustrated. Lea & Febiger, Publishers, Philadelphia and New York. 1910. (Cloth, $4.75 net.)

The author of this work is prepared to give information on the subject of general practice, by reason of a long apprenticeship and finally earning a professorship in one of the largest institutions of medical education in this country. Moreover, as chief bacteriologist to a large hospital he laid a firm foundation for the post of teacher in medicine. Therefore, we may assume that Kelly is well prepared to teach and write on the subject of the practice of medicine. This treatise is prepared specially for the student and junior physician to instruct them, as the author himself so well states, as to the nature, discrimination, and management of disease. The arrangement of the work is unusual and claims attention on that account, as well as for its other meritorious methods. Its first section takes up infectious diseases, more or less exhaustively dealt with according to their importance; then in section two come the intoxications, handled briefly; the disorders of metabolism form the subject matter of the third section; diseases of the ductless glands and of internal secretion are presented in the fourth section; next come in the fifth section, diseases of the blood and hemopoietic system; the sixth section deals with diseases of the circulatory system; the seventh, with diseases of the respiratory system; the eighth, with diseases of the digestive system; the ninth, with diseases of the urinary system; the tenth, with diseases of the nervous system; the eleventh, with diseases of the muscles; and the twelfth and final section, with diseases of the bones and joints.

From this it will appear that the treatise is an exhaustive exposition of the present science of medical practice. The cyclopedic textbooks, of which there are several, rest with dignity on the library shelves as works of reference, but, the student and the younger practitioner need just such a compact, single-volume book as this to aid, to guide him over the rough and stormy seas of everyday professional life. We regard it as one of the better of this class of works, and commend it not only to the student and junior physician, but feel sure everyone in active medical practice will find it a safe exponent of present day medicine.

